# *ssb* Gene Duplication Restores the Viability of Δ*holC* and Δ*holD Escherichia coli* Mutants

**DOI:** 10.1371/journal.pgen.1004719

**Published:** 2014-10-16

**Authors:** Stéphane Duigou, Maud Silvain, Enrique Viguera, Bénédicte Michel

**Affiliations:** 1 Centre de Génétique Moléculaire, Centre National de la Recherche Scientifique, Gif-sur-Yvette, France; 2 Plateforme Intégrée IMAGIF, Centre National de la Recherche Scientifique, Gif-sur-Yvette, France; 3 Área de Genética, Facultad de Ciencias, Universidad de Málaga, Málaga, Spain; Uppsala University, Sweden

## Abstract

The HolC-HolD (χψ) complex is part of the DNA polymerase III holoenzyme (Pol III HE) clamp-loader. Several lines of evidence indicate that both leading- and lagging-strand synthesis are affected in the absence of this complex. The *Escherichia coli* Δ*holD* mutant grows poorly and suppressor mutations that restore growth appear spontaneously. Here we show that duplication of the *ssb* gene, encoding the single-stranded DNA binding protein (SSB), restores Δ*holD* mutant growth at all temperatures on both minimal and rich medium. RecFOR-dependent SOS induction, previously shown to occur in the Δ*holD* mutant, is unaffected by *ssb* gene duplication, suggesting that lagging-strand synthesis remains perturbed. The C-terminal SSB disordered tail, which interacts with several *E. coli* repair, recombination and replication proteins, must be intact in both copies of the gene in order to restore normal growth. This suggests that SSB-mediated Δ*holD* suppression involves interaction with one or more partner proteins. *ssb* gene duplication also suppresses Δ*holC* single mutant and Δ*holC* Δ*holD* double mutant growth defects, indicating that it bypasses the need for the entire χψ complex. We propose that doubling the amount of SSB stabilizes HolCD-less Pol III HE DNA binding through interactions between SSB and a replisome component, possibly DnaE. Given that SSB binds DNA *in vitro* via different binding modes depending on experimental conditions, including SSB protein concentration and SSB interactions with partner proteins, our results support the idea that controlling the balance between SSB binding modes is critical for DNA Pol III HE stability *in vivo*, with important implications for DNA replication and genome stability.

## Author Summary

Both replication polymerases and single-stranded DNA binding proteins (SSB, which associate with single-stranded DNA exposed transiently during replication) are ubiquitous and show high levels of functional and structural conservation across all species. Among the nine different polypeptides that compose the bacterial replicative polymerase, the HolC-HolD (χψ) complex interacts with SSB, and is crucial for normal growth in the model bacteria *Escherichia coli*. Interestingly, many bacterial species lack this complex, where its function is presumably carried out by other polymerase components. With the aim of better understanding HolC-HolD (χψ) complex function in *E. coli*, we isolated growth defect suppressor mutations of the *holD* mutant. We found that *ssb* gene duplication and the consequent doubling of SSB protein expression, renders the entire χψ complex dispensable for growth. We also show that growth-defect suppression requires the presence of the SSB C-terminal amino acids in both *ssb* gene copies. This C-terminal tail promotes interaction between SSB and its partner proteins. Thus, our results indicate that *in vivo* SSB concentration plays a key role in maintaining polymerase stability and replication efficiency, in a reaction that involves SSB interactions with protein partner(s) other than χψ.

## Introduction

Chromosome replication is performed by the replisome, a molecular machine present in all living organisms with strong structural and functional similarities [1]–[4]. Replisomes combine the action of a primosome and a polymerase, for which the enzymes from *Escherichia coli* have proved an invaluable model for understanding their function. The *E. coli* primosome is itself composed of two interacting enzymes, the hexameric DnaB helicase that opens double-stranded DNA and the DnaG primase that synthesizes leading- and lagging-strand primers. DNA is synthesized by the DNA polymerase III holoenzyme (Pol III HE), composed of polypeptides encoded by 9 different genes. The holoenzyme is composed of three core polymerases [5], each made up of a polymerase α subunit (encoded by *dnaE*), a proofreading ε subunit (*dnaQ*), and a θ stability factor (*holE*). DNA binding by leading- and lagging- strand core polymerases is stabilized through interactions with the β-clamp (*dnaN*). Lagging-strand synthesis is discontinuous and Okazaki fragments (OF) are joined by the ligase. The role of the third core polymerase is still under investigation; current models suggest that it replaces the lagging-strand polymerase when needed [6]. β-clamps are loaded onto DNA for replication initiation and for the synthesis of each OF by a complex called the clamp loader. The minimal clamp loader core is a pentameric complex containing a δ (*holA*), a δ′ (*holB*) and three τ (*dnaX*) protein subunits. A χψ complex (*holC*, *holD*) connects this pentameric complex to DNA, as ψ (HolD) interacts with τ [7], [8] and χ (HolC) interacts with the single-stranded DNA binding proteins (SSB) that cover the lagging-strand template [9]–[12]. The three-dimensional structure of the χψ complex has been determined, identifying the sites of interaction between ψ and τ, and between χ and SSB [12], [13]. *In vitro* each clamp loader complex contains a single χψ complex, but four may be associated with the replisome *in vivo* [4], [5], [8]. How the three additional *in vivo* χψ complexes are organized is currently unknown.

The clamp loader complex ensures replisome cohesion through interactions between τ and Pol III, τ and the DnaB helicase, and χ and SSB [4]. Clamps and clamp-loaders are universally conserved in structure and function, for example PCNA and RFC, respectively, in eukaryotes [2]. In contrast, ψ and χ have only been found in proteobacteria [1], [14], [15] and no homologous proteins have been reported in eukaryotes. Using *E. coli* mutants to analyze the role of the χψ complex provides an alternative approach toward understanding chromosome replication and the molecular mechanisms that underlie clamp-loader function.

*In vitro* comparison of clamp loading and replication in the presence and absence of χψ have led to the identification of three main putative functions. Firstly, ψ-τ interactions stabilize the clamp loader complex, allowing it to form even at the low protein concentrations found *in vivo* [16]. The presence of ψ alone increases clamp-loader ATPase activity, and its affinity for DNA and the β-clamp [8], [17]. Secondly, in the presence of SSB, owing to χ-SSB interactions, χψ increases the affinity of the clamp-loader complex to primer-template DNA, stimulates clamp loading activity and increases Pol III processivity [9], [18]. Thirdly, χψ also promotes the *in vitro* displacement of primase from RNA primers, by switching from primase-SSB to χ-SSB interactions at the primer-template junction, and thus participating in primase recycling at replication forks [19].

The above properties indicate that χψ mainly acts on the lagging-strand template, which is SSB-coated and subject to clamp loading every 1–2 seconds. Accordingly, a *holC* deletion confers a hyper-recombination phenotype that can be explained by defective lagging-strand synthesis [20]. Moreover, a *holD* mutant was isolated in a screen for hyper-recombination mutants [21]. On the other hand, several lines of *in vivo* and *in vitro* evidence suggest that decreasing the cellular level of χ or ψ proteins affects both lagging- and leading-strand synthesis. A *holD* point mutation was shown to trigger replication fork reversal, which is caused by replication fork arrest and the subsequent annealing of leading- and lagging-strand ends to form a Holliday junction adjacent to a double-stranded DNA end [21], [22]. In addition, a mutation affecting *holC* suppressed the growth defects caused by replication over-initiation, possibly because it slows down replication fork progression [23]. Finally, *in vitro* studies using χ variants with impaired SSB interaction capacity revealed defects in leading-strand synthesis and resulted in the production of shorter OFs, in agreement with the idea that *holC* and/or *holD* impairment affects the synthesis of both strands during replication [12].

Deletion of the *holD* gene strongly affects *E. coli* growth at 30°C and is lethal at higher temperatures. These defects could be partially suppressed by blocking the SOS response [24]. The *E. coli* SOS response is triggered by the accumulation of RecA-coated single-stranded DNA (ssDNA) followed by proteolysis of the LexA repressor, inducing the expression of more than 40 LexA-repressed DNA repair genes [25], [26]. We have previously shown that the SOS-induced DinB and Pol II bypass polymerases are responsible for the deleterious effects of the SOS response on Δ*holD* mutant growth [24]. We proposed that a combination of replisome destabilization in the absence of ψ and displacement of the destabilized replisomes by these two SOS-induced polymerases was, at least in part, responsible for the poor growth of the Δ*holD* mutant [24].

In this work, we characterize a spontaneous suppressor mutation of the Δ*holD* growth defects and identify it as an *ssb* gene duplication. We find that this duplication also suppresses the growth defects of a Δ*holC* mutant but does not prevent Δ*holD*-induced expression of SOS response genes. We propose that *ssb* gene duplication directly compensates for the absence of the χψ complex by stabilizing the association of Pol III HE to DNA.

## Results

### Characterization of a Δ*holD* mutant growth defect suppressor

**Figure 1:**
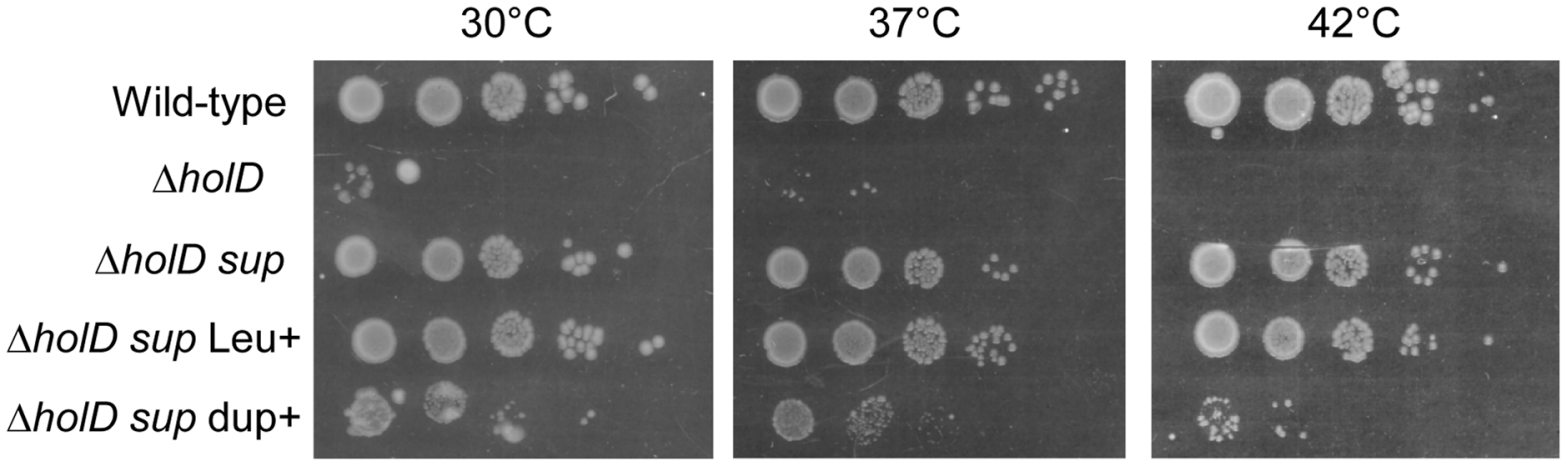
A 10 kb duplication restores Δ*holD* viability. In a first step isolated colonies were obtained by plating on MM appropriate dilutions of overnight cultures or, for strains containing pAM-*holD*, appropriate dilutions of cultures propagated for 8 hours in the absence of IPTG, and incubating plates for 3 days at 30°C. Isolated colonies were suspended in 1 ml of MM salt medium. Serial 10-fold dilutions (10^−2^ to 10^−6^) were made and 7 µl drops of each dilution were spotted on three MM plates incubated overnight at 37°C or 42°C or for two days at 30°C. Wild-type, JJC2069; Δ*holD*, JJC2067 cured of pAM-*holD*; Δ*holD sup*, JJC2394; Δ*holD sup* Leu^+^, JJC6178; Δ*holD sup* dupΔ, JJC6217 cured of pAM-*holD*.

**Figure 2:**
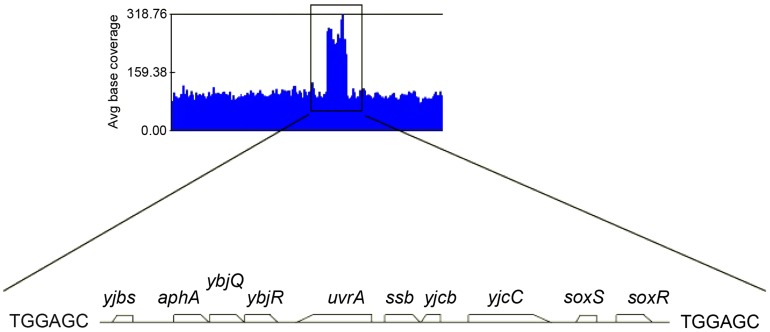
Duplicated sequence in JJC2394. Schematic representation of several JJC2394 sequence reads aligned to the wild-type *E.coli* genome using GenomeStudio Software (Illumina). The black box corresponds to a duplication of the 10 kb chromosome region shown below it. A 6 bp repeated sequence was identified at the duplication boundaries.

Because the Δ*holD* mutant is slow growing and accumulates suppressor mutations, we propagated it in the presence of a complementing plasmid that replicates from a conditional origin. The pAM-*holD* plasmid carries the wild-type *holD* gene and only replicates in the presence of the *lac* inducer isopropyl β-D thio-galactoside (IPTG) [24]. This plasmid was then cured prior to each experiment to restore the Δ*holD* mutant condition. Depending on the experiment, we either generated mixed cultures containing at least 99% cured cells by growing Δ*holD* [pAM-*holD*] cells in the absence of IPTG for about 15 generations, or isolated plasmid-less colonies by plating Δ*holD* [pAM-*holD*] cultures propagated in the absence of IPTG for 8 hours on IPTG-free medium. Cultures or colonies were confirmed to be free of both complementing plasmids and suppressor mutations. All strains used in this work carry a *sfiA* mutation (*sfiA*::MudAp*lacZ* or *sfiA11*) to prevent SfiA-mediated cell division blockage upon SOS induction. When grown at 30°C on minimal medium supplemented with casamino acids (hereafter MM), Δ*holD* cells formed rare small colonies together with rapidly-growing colonies that we suspected to have acquired a suppressor mutation (Figure 1, Figure S1, Figure S3, [24]). Putative Δ*holD* suppressing clones were able to form normal sized colonies over two days when grown at 30°C on MM. We sequenced the genome of one such clone, designated JJC2394, and compared its sequence to that of the parental Δ*holD* mutant. Two mutations were found in JJC2394: a *leuO* point mutation (A233V) and a 10 kb duplication (Figure 2). Reversing the *leuO* mutation to Leu^+^ by P1 transduction did not affect JJC2394 viability. In contrast, removing the duplication abolished most of the suppression effect, indicating that it is important for the suppressor phenotype (Figure 1). The duplication is flanked by 6 base pairs (bp) microhomology DNA sequences and lies between positions 4 266 351 and 4 276 297 on the *E. coli* MG1655 chromosome used as the reference strain (Figure 2). The duplicated region contains 10 genes: five of unknown function, *aphA* encoding a periplasmic phosphatase, *uvrA* encoding a nucleotide excision repair protein, *soxS* and *soxR* encoding the superoxide response activator, and *ssb*.

### Duplication of the *ssb* gene suppresses Δ*holD* mutant growth defects

**Figure 3:**
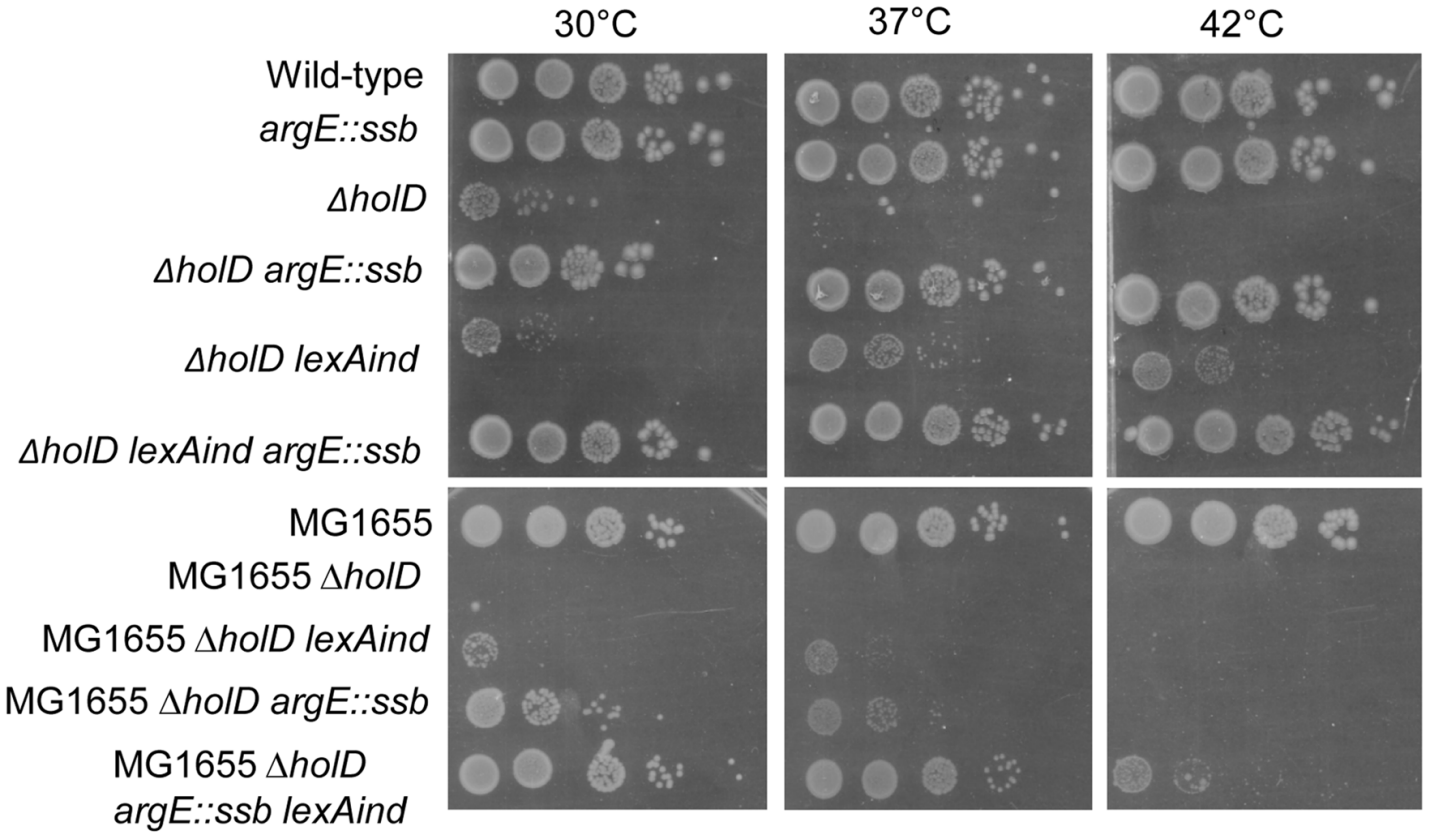
In an AB1157 background, a *ssb* gene duplication is sufficient to suppress Δ*holD* mutant growth defects. Serial dilutions of suspended colonies were spotted on MM and incubated as described in the Figure 1 legend. Top panel: Wild-type, JJC2069; *argE::ssb*, JJC6047; Δ*holD*, JJC6050 cured of pAM-*holD*; Δ*holD argE::ssb*, JJC6110; Δ*holD lexAind*, JJC1524 cured of pAM-*holD*; Δ*holD lexAind argE::ssb*, JJC6077 cured of pAM-*holD*. Bottom panels: MG1655, JJC3523; MG1655 Δ*holD*, JJC6363 cured of pAM-*holD*; MG1655 Δ*holD lexAind*, JJC6420 cured of pAM-*holD*; MG1655 Δ*holD argE::ssb*, JJC6394 cured of pAM-*holD*; MG1655 Δ*holD argE::ssb lexAind*, JJC 6419 (spontaneously cured of pAM-*holD* during construction at 30°C).

Since *ssb* is the only one of the duplicated genes directly involved in DNA replication, we hypothesized that the presence of two copies of *ssb* might be responsible for the suppressor phenotype. To test this idea, we constructed a strain carrying an additional copy of *ssb* inserted into the *argE* locus, which is located approximately 120 kb from *ssb*. Strains harboring the *argE*::*ssb* insertion carry the same number of *ssb* genes as the *ssb* tandem duplication throughout the cell cycle, and the two *ssb* copies are stably maintained due to the distance separating them. Western analysis using anti-SSB antibodies showed that JJC2394 cells and cells containing the *argE*::*ssb* insertion expressed two to three times as much SSB protein as wild-type cells (Figure S2). Although over-production of SSB from a plasmid affects the growth of wild-type *E. coli* and induces the SOS response [27], *ssb* gene duplication appears to have no deleterious effect since *argE*::*ssb* did not affect the growth of wild-type cells and did not induce the SOS response (Figure 3, Table 1).

**Table 1:** SOS induction in Δ*holD* and Δ*holD* suppressed mutants.

Strain	genotype	β-gal Miller Units 30°C	β-gal Miller Units 42°C 3 hr	N 30°C/42°C
JJC2069	wild-type	74±11	59±9	11/9
JJC2394	Δ*holD sup*	119±29	225±24	12/10
JJC6047	*argE::ssb*	57±9	37±7	5/5
JJC2067S	Δ*holD*	230±15	334±39	8/10
JJC2068S	Δ*holD recF*::Tn*5*	104±14	96±3	8/8
JJC6161	Δ*holD argE::ssb*	180±25	420±52	32/18
JJC6128	Δ*holD argE::ssb*	170±16	369±46	16/13
JJC6180	Δ*holD argE::ssb recF*::Tn*5*	112±8	152±20	12/12
JJC6060	Δ*holD sup recF*::Tn*5*	79±12	82±9	8/8
JJC6216	Δ*holD sup dup*Δ *argE::ssb*	115±24	201±14	10/10
JJC6162	*argE::ssb*ΔC5	53±11	37±4	14/12

N indicates the number of independent experiments. Since JJC2067 isolated colonies could not be used, JJC2067 and JJC2068 overnight cultures propagated in MM lacking IPTG were diluted 50 fold and grown in MM for the experiment.

Next, we analyzed the growth properties of Δ*holD argE*::*ssb* cells. The presence of two *ssb* gene copies restored wild-type levels of colony formation to Δ*holD* mutants when grown on MM and LB, at 30°C, 37°C and 42°C (Figure 3, Figure S3). In fact, although the *lexAind* mutation allowed Δ*holD* cells to be propagated in liquid cultures [24], Δ*holD lexAind* colonies were slower growing and reduced in number compared to Δ*holD argE::ssb* (Figure 3, Figure S3).

### Duplication of the *ssb* gene does not prevent SOS response induction in the Δ*holD* mutant

As preventing SOS induction improves Δ*holD* mutant growth [24], we tested whether the *ssb* duplication acts by decreasing SOS-induction. SOS expression was measured using *lacZ* under the control of the SOS-induced promoter *sfiA* in Δ*holD* mutants and in suppressed strains. β-galactosidase expression was measured in cells propagated either at 30°C or after shifting to 42°C for 3 hours (Table 1). As reported previously, the Δ*holD* mutation induces the SOS response at both temperatures and this induction is prevented by *recF* inactivation [24]. In the JJC2394 spontaneous-suppression mutant, the SOS response was decreased compared to the Δ*holD* mutant at 30°C and 42°C, but was still significantly higher than in wild-type cells. The Δ*holD argE*::*ssb* mutant exhibited a similar SOS response to that of the Δ*holD* single mutant, showing that the suppression phenotype conferred by *ssb* duplication is not a consequence of SOS inactivation. SOS expression in Δ*holD argE*::*ssb* cells and in JJC2394 was largely RecF-dependent (Table 1), in the same way as in Δ*holD* cells. RecF, RecO and RecR proteins specifically promote RecA loading onto ssDNA gaps. Thus, RecF dependence suggests that the SOS response in Δ*holD* cells is induced by the accumulation of ssDNA gaps, possibly formed during lagging-strand synthesis. Inactivating the SOS response with a *lexAind* mutation did not further improve the capacity of Δ*holD argE*::*ssb* cells to form colonies at different temperatures (Figure 3, Figure S3). This is consistent with the fact that SOS induction is not required for Δ*holD argE*::*ssb* cell viability and that the viability of this double mutant is equivalent to wild-type bacteria.

For historical reasons, this work was realized in an AB1157 background. In the more commonly used MG1655 strain background, suppression of Δ*holD* growth defects by *ssb* gene duplication was observed at 30°C and 37°C but not at 42°C (Figure 3). In this case, introducing the *lexAind* mutation slightly improved Δ*holD* growth. Interestingly *lexAind* and *argE::ssb* suppressor mutations had additive effects on MG1655 Δ*holD* viability, with the MG1655 Δ*holD lexAind argE::ssb* mutant exhibiting similar plating efficiency to that of wild-type MG1655 at 30°C and 37°C. Nevertheless, in contrast to the AB1157 background, colonies were smaller than wild-type at 37°C and this strain was unable to propagate at 42°C (Figure 3).

The higher SOS response levels in Δ*holD argE*::*ssb* cells compared to the Δ*holD sup* cells of JJC2394 suggested the presence of an additional mutation in the latter strain (Table 1). However, the mutation(s) responsible for this difference could not be identified from the chromosome sequence. *lexA* and recombination genes were intact and, accordingly, survival to UV irradiation was unaffected in JJC2394 (Figure S4). To test the effect of the 10 kb tandem duplication, the *argE*::*ssb* allele was introduced into JJC2394 resulting in the spontaneous loss of the duplication, presumably by homologous recombination. The resulting strain carried the *argE*::*ssb* allele instead of the spontaneous 10 kb duplication but was otherwise identical to JJC2394. Significantly, the new strain showed the same SOS response levels as the Δ*holD sup* strain JJC2394 rather than the reconstructed Δ*holD argE*::*ssb* strain (Table 1, JJC6216). Therefore, the lower expression of the SOS response in JJC2394 compared to Δ*holD argE*::*ssb* is not caused by another gene within the 10 kb duplication and remained unexplained.

### A ∼10-fold increase in DinB expression is not detrimental to Δ*holD argE*::*ssb* cell growth

**Figure 4:**
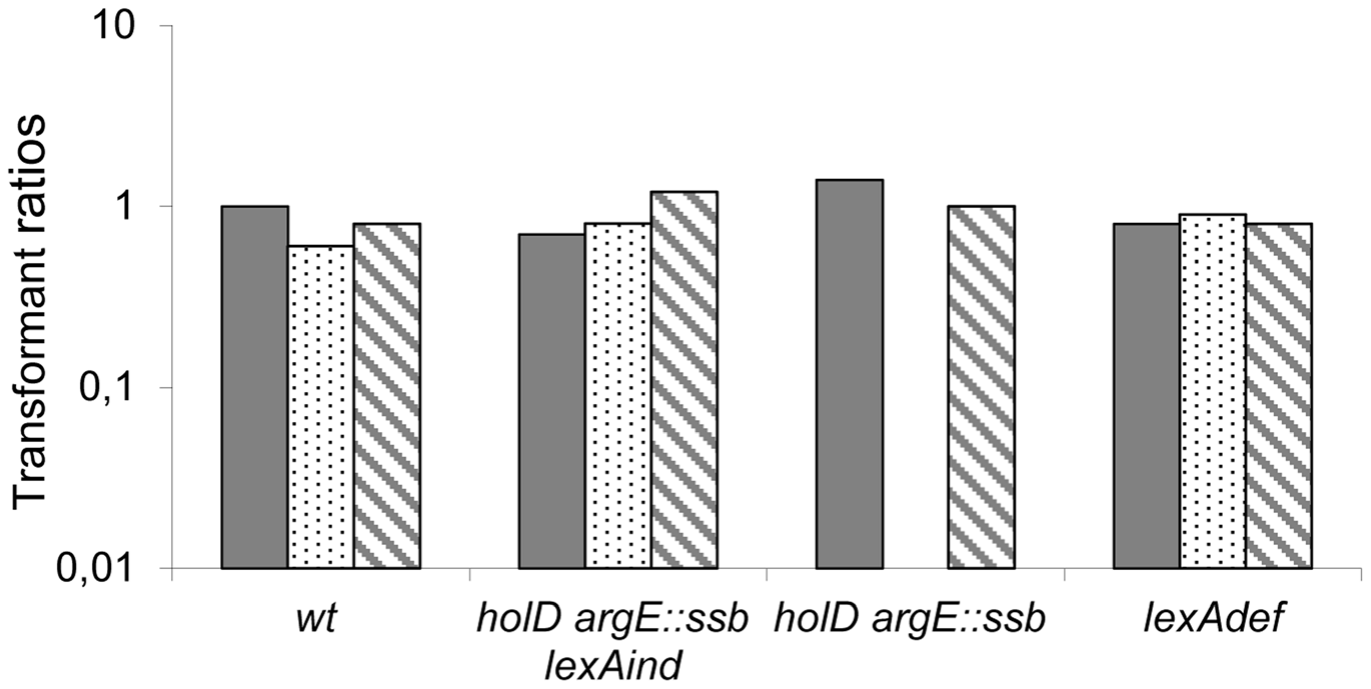
pGB-*dinB* is lethal to Δ*holD argE::ssb* only when the SOS response is induced. Plain, dotted and hatched boxes correspond to pGB2, pGB-*dinB* and pGB-*dinB*ΔC5 transformation efficiencies, respectively, when grown on MM after overnight incubation at 37°C, normalized to pGB2 transformation efficiency on LB at 37°C in the same strain (see Table S2 for transformation efficiencies on LB). Wild-type (wt) JJC40 and JJC1945; Δ*holD argE::ssb lexAind*, JJC6133; Δ*holD argE::ssb*, JJC6110, *lexA*Def JJC6488. Averages of three to four experiments are shown. pGB-*dinB* transformants appeared on MM at 37°C in two days in one JJC6110 (Δ*holD argE::ssb*) experiment out of four, and in the same way as clones obtained on LB (Figure S5), they could not be propagated.

We previously showed that inactivating either of the *dinB* and *polB* SOS-induced polymerase genes improves Δ*holD* mutant viability at 37°C, while inactivating both restored growth at 42°C [24]. These results suggested that DinB and PolB polymerases participate in the destabilization of HolD-less Pol III HE upon SOS response induction. One possibility is that the restoration of Δ*holD* mutant growth by *ssb* duplication is linked to the destabilizing effect of SOS-induced DinB on Pol III HE. DinB levels increase about 8- to 10-fold upon SOS induction [25], [28], [29], and increase to similar levels when expressed from a pSC101-derived vector [29]. Thus, we used the pSC101-derived vector pGB2 to compare the effects of increased expression of the wild-type *dinB* gene (pGB-*dinB*). When tested in a *lexAind* background to prevent SOS induction, the transformation efficiency of [pGB-*dinB*] was similar for wild-type and Δ*holD argE*::*ssb* mutant bacteria in all conditions, confirming that 8-fold over-production of DinB is not deleterious to the Δ*holD argE*::*ssb* mutant (JJC6133 Figure 4, Table S2 and Figure S5). Moreover, pGB-*dinB* was not deleterious for growth in HolD^+^ LexA^+^ or LexAdef backgrounds, confirming previous results showing that DinB expressed from a pSC101 replicon is not deleterious for growth, even in the absence of the LexA repressor [29]. In these conditions, DinB is expressed at 8- and 30-times the wild-type chromosomal level, respectively [29], and replication in wild-type cells is only sensitive to the higher levels of DinB over-expression [30], [31]. However, pGB-*dinB* could not be introduced into Δ*holD argE*::*ssb* or JJC2394 cells on MM (Figure 4, Table S2); on LB, Δ*holD argE*::*ssb* [pGB-*dinB*] clones were obtained at 37°C and 42°C but could not be propagated (Figure S5). These results suggest that *ssb* gene duplication does not stabilize ψ-less Pol III HE enough to compensate for the effect of pGB plasmid-mediated DinB overexpression combined with SOS response activation.

To explore the mechanism behind this effect, we performed the same assay using a *dinB* gene lacking 5 C-terminal amino acids required for interaction with the β-clamp (pGB-*dinB*ΔC5 [32]). The absence of detrimental effects following expression of this deletion mutant (pGB-*dinB*ΔC5, Figure 4, Table S2 and Figure S5) demonstrates that a functional interaction between DinB and DnaN is required for the effects of 30-fold DinB overproduction. Moreover, it shows that this interaction induces the substitution of β-clamp DnaN-bound Pol III by DinB.

Altogether, these results indicate that doubling SSB concentration *in vivo* protects the Δ*holD* mutant against the deleterious effects of a 8- to 10-fold increase in DinB, regardless of whether this increase is caused by SOS induction, or increased *dinB* gene expression from a ∼10 copy-number plasmid in the absence of SOS induction. These results suggest that doubling the amount of SSB stabilizes Pol III HE DNA binding in the absence of HolD and consequently improves resistance to physiological increases in DinB levels, such as those produced by SOS induction. However, the Δ*holD argE*::*ssb* mutant remains sensitive to ∼30-fold increases in DinB production, showing that even in the presence of twice the normal amount of SSB, the HolD-less Pol III HE complex is more sensitive than the wild-type holoenzyme to non-physiological DinB amounts.

### A five amino acid *ssb* C-terminal deletion prevents *ssb*-mediated suppression of Δ*holD* growth defects

**Figure 5:**
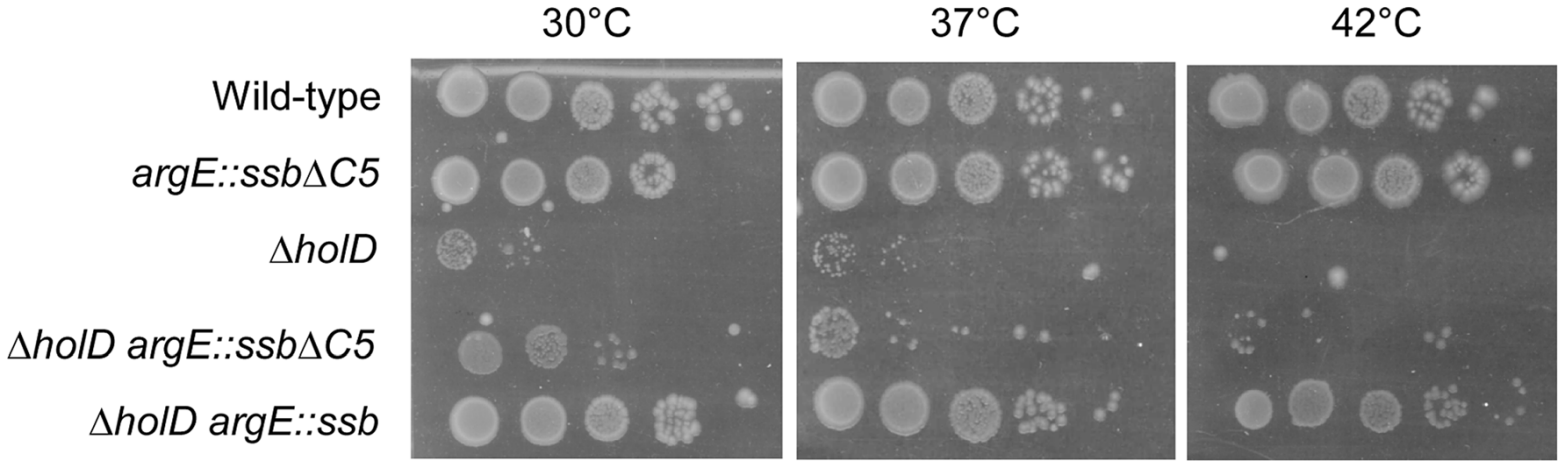
The five C-terminal amino acids of SSB are required for growth suppression. Serial dilutions of suspended colonies were spotted on MM and incubated as described in the Figure 1 legend. Wild-type, JJC2069; *argE::ssb*Δ*C5*, JJC6162; Δ*holD*, JJC2067 cured of pAM-*holD*; Δ*holD argE::ssb*Δ*C5*, JJC6078 cured of pAM-*holD*; Δ*holD argE::ssb*, JJC6076 cured of pAM-*holD*.

SSB interacts with a large number of DNA replication, recombination and repair proteins via its C-terminus in both *E. coli* and *Bacillus subtilis* (reviewed in [33], [34]). In order to test whether these interactions play a role in the suppression of Δ*holD* defects by *ssb* duplication, we constructed a strain in which the additional copy of *ssb* inserted into *argE* contains a five amino acid C-terminal deletion (*ssb*-ΔC5; see Materials and Methods). The *argE*::*ssb*-ΔC5 allele did not affect wild-type growth and did not induce the SOS response (Figure 5, Table 1), showing that expression of this SSB truncated protein does not affect the function of the wild-type protein. Growth of the Δ*holD argE*::*ssb*-ΔC5 mutant was tested on MM and on LB at different temperatures. Compared to the Δ*holD* single mutant, Δ*holD argE*::*ssb*-ΔC5 was only slightly more viable on MM at 30°C and rapidly acquired suppressor mutations (Figure 5, Figure S3). We conclude from these experiments that Δ*holD* mutant growth defects can only be suppressed by an additional copy of *ssb* carrying an intact C-terminus. This result suggests that interaction(s) with SSB partner(s) are crucial for the rescue of Δ*holD* mutant by increased *ssb* gene dosage.

### *ssb* duplication suppresses Δ*holC* and Δ*holC* Δ*holD* mutant growth defects

The χψ complex (HolC-HolD) bridges the minimal clamp loader complex to SSB. We hypothesized that χ (HolC) might be the SSB interacting protein required for Δ*holD* growth defect suppression. If doubling the amount of SSB allows χ to act without ψ, introduction of Δ*holC* should abolish the suppression. Alternatively, if *ssb* gene duplication bypasses the need for the entire χψ complex, it should also suppress the growth defects of Δ*holC* and Δ*holC* Δ*holD* mutants. We tested these ideas using the Δ*holC102*::Cm^R^ deletion mutant [20].

**Figure 6:**
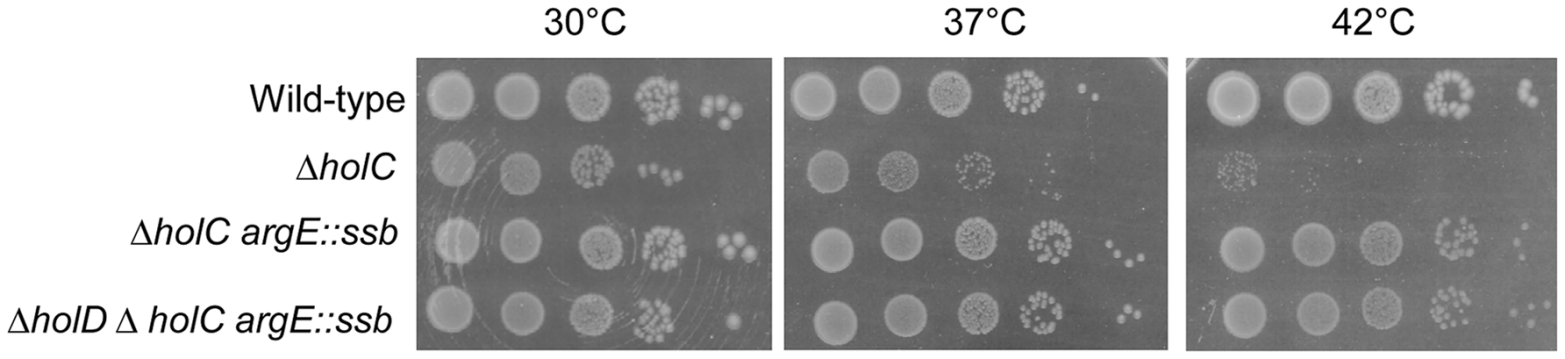
*ssb* gene duplication restores normal growth to Δ*holC* and Δ*holC* Δ*holD* mutants. Serial dilutions of suspended colonies were spotted on MM and incubated as described in the Figure 1 legend. Wild-type, JJC1945; Δ*holC*, JJC6469 cured of pAM-*holC*; Δ*holC argE::ssb*, JJC6476 cured of pAM-*holC*; Δ*holD* Δ*holC argE::ssb*, JJC6470 cured of pAM-*holC*.

Growth of the Δ*holC* mutant was strongly affected at 42°C and only slightly affected at 30°C and 37°C, both on MM and LB (R. Maurer personal communication, Figure 6 and Figure S6). It is worth noting that Δ*holC* is less deleterious for growth at 30°C and 37°C than Δ*holD*, suggesting a role for ψ-τ (HolD-DnaX) interaction in Pol III HE stability. We constructed pAM-*holC* and pAM-*holCD* plasmids that carry wild-type copies of *holC* or both *holC* and *holD* genes respectively, which were cured at the onset of each experiment (see Materials and Methods). We analyzed Δ*holC* single, Δ*holC argE::ssb* double and Δ*holC* Δ*holD argE::ssb* triple mutants (Figure 6 and Figure S6) and obtained similar results regardless of whether the strains were originally constructed in the presence of pAM-*holC* or pAM-*holCD*. The *ssb* gene duplication conferred viability to both Δ*holC* single and Δ*holC* Δ*holD* double mutants at all temperatures, although at 42°C Δ*holC argE::ssb* and Δ*holC* Δ*holD argE::ssb* colonies were slightly smaller than wild-type. Thus, doubling the amount of SSB suppresses growth defects caused by the absence of the entire χψ complex, regardless of whether χψ function is affected by the inactivation of *holC*, *holD* or both genes. We conclude that *ssb* duplication suppresses the growth defects caused by a HolCD-less Pol III holoenzyme via SSB interactions with a replisome protein other than χ.

## Discussion

In this work, we show that *ssb* gene duplication restores the viability of Δ*holD* cells at all temperatures. Since the SOS response remains induced in Δ*holD argE::ssb* cells, *ssb* gene duplication renders the HolD-less Pol III holoenzyme insensitive to SOS-induced levels of DinB and Pol II proteins. This observation suggests that doubling the amount of SSB stabilizes HolD-less Pol III HE DNA binding at replication forks, which increases its resistance to competing SOS-induced polymerases. Suppression was only observed when both *ssb* gene copies were intact and not if the second *ssb* copy carried a five C-terminal amino acid deletion. These results suggest that suppression bypasses the entire χψ complex and requires SSB interaction with a replisome partner other than χ.

### Δ*holD* mutant growth defects are mainly caused by the intrinsic instability of HolD-less Pol III holoenzyme DNA binding

*In vivo*, the Δ*holD* mutant accumulates gaps, as deduced from RecF-dependent constitutive SOS expression, and suffers from replication arrest and polymerase loss, as deduced from the occurrence of replication fork reversal and from its sensitivity to SOS-induced polymerases [21], [24]. Accordingly, purified χ proteins containing a mutation that specifically affects SSB interaction were clearly impaired for both leading- and lagging-strand synthesis and for replisome stability [12]. The well-documented importance of χψ for lagging-strand synthesis *in vitro* [16], [17], [19] suggests that the gaps that induce the SOS-response *in vivo* are formed on the lagging strand. We observe that *ssb* gene duplication restores the viability of Δ*holD* mutant cells but does not prevent RecF-dependent SOS induction, thus does not suppress gap formation. Therefore, excessive gap formation is not directly responsible for the poor viability of the Δ*holD* mutant. Furthermore, *ssb* duplication restores normal Δ*holD* growth in the presence of an 8 to 10-fold excess of DinB, expressed either from the SOS-induced chromosomal copy or from a low copy plasmid in the absence of SOS induction. Consequently, we propose that an intrinsic lack of stability of HolD-less Pol III HE bound to DNA is responsible for the growth defects of the Δ*holD* mutant, and that *ssb* gene duplication acts by stabilizing the HolD-less Pol III holoenzyme. It is worth noting that competition by SOS-induced polymerases is not the only reason for HolD-less Pol III HE instability, as *lexAind* mutation does not suppress Δ*holD* growth defects as efficiently as *ssb* gene duplication.

In the MG1655 background, suppression of Δ*holD* growth defects by either *lexAind* or *ssb* duplication is partial, showing that the effects of these two suppressor mutations are additive. We propose that a combination of decreased expression of SOS-induced polymerases (*lexAind*) and increased Pol III HE DNA stability (*ssb* duplication) is necessary to restore viability in this background. In AB1157, where *ssb* duplication suppresses Δ*holD* growth defects quite efficiently, the additive effects of *lexAind* and *ssb* duplication are not directly detectable. The thermosensitivity of the Δ*holD argE::ssb lexAind* MG1655 mutant, also observed for the Δ*holC* AB1157 single mutant, is interesting since no protein is intrinsically sensitive to high temperature in these mutants. It cannot be accounted for solely by a higher number of replication forks per chromosome at 42°C compared to 30°C, since the number of replication forks per chromosome is also increased in rich medium and these mutants show no rich medium sensitivity. High temperature affects protein-protein and protein-DNA interactions and the sensitivity of these mutants to high temperature supports the idea that the primary defect of the HolCD-less Pol III holoenzyme is its intrinsic instability on DNA. In agreement with a direct role for HolD in clamp loader complex stability *in vitro* [8], [17], growth is clearly more affected at both 30°C and 37°C in Δ*holD* mutant cells than in cells lacking *holC*.

### *ssb* duplication stabilizes the HolCD-less Pol III holoenzyme

*In vitro* SSB binds ssDNA in multiple binding modes, among which the two major forms are (SSB)_35_ and (SSB)_65_, where 35 and 65 nucleotides, respectively, are wrapped around a SSB tetramer [35]. SSB proteins are also mobile on ssDNA, undergoing random diffusion along ssDNA mainly in the (SSB)_65_ binding mode (Zhou et al. 2011). The (SSB)_35_ binding mode is less mobile, more stable, and highly cooperative, forming protein clusters or filaments on DNA [35]–[37]. The binding mode is determined by salt concentration and by the SSB protein to ssDNA ratio. Increasing SSB concentration *in vitro* shifts the binding mode toward the (SSB)_35_ form [36]. It has been proposed that the binding mode could also be influenced by protein interactors, and actually interaction between PriC and the C-terminal tail of SSB can also shift the ssDNA binding mode from (SSB)_65_ to (SSB)_35_ [38]. The primary effect of *ssb* gene duplication and the resulting increase in SSB protein concentration could involve a shift from the (SSB)_65_ to the (SSB)_35_ binding mode on the lagging-strand template at *in vivo* salt concentration. This phenomenon could compensate for the absence of the χψ complex if χ-SSB interaction is normally responsible for the shift, as has been hypothesized [36]. Nevertheless, it should be noted that to date the existence of different SSB binding modes, and their dependence on SSB concentration and on SSB-protein interactions have only been demonstrated *in vitro* and remain to be tested *in vivo*.

A C-terminal SSB truncation promotes ssDNA binding and shifts the equilibrium toward the (SSB)_35_ mode *in vitro* [10], [36], [39], [40]. Therefore, it is unlikely that this deletion prevents a putative shift from (SSB)_65_ to (SSB)_35_ in cells expressing both wild-type and truncated SSB. Thus, the requirement for two intact *ssb* genes to suppress Δ*holD* mutant growth defects may instead reflect a need for SSB interaction(s) with one or more protein partner(s) [33]. Three replisome proteins have been reported to interact with SSB: χ, primase and the α polymerase (DnaE) [33], [41]. χ is not required for suppression since Δ*holC* and Δ*holC* Δ*holD* mutants are also fully suppressed by the *ssb* gene duplication. The SSB and the primase appear to interact via SSB C-terminal amino acids and a specific region of the primase [42]. Primase could be a key SSB interacting protein for stabilization of the HolD-less Pol III holoenzyme, although its requirement for the OF synthesis and the high level of SOS induction in Δ*holD argE*::*ssb* cells suggests that gap formation during OF synthesis is not suppressed by *ssb* gene duplication. The SSB-DnaE interaction was detected in a Tap-Tag high-throughput analysis of *E. coli* proteins using DnaE as bait and SSB as prey [41]. Even though the protein regions involved in SSB and DnaE interaction have not yet been identified, this interaction could also be crucial for the growth of HolD-less Pol III containing cells. Interaction between the SSB C-terminus and a Pol III holoenzyme component other than χ has been shown to stimulate initiation complex formation [43]. In cells lacking χψ, DnaE-SSB interaction could be needed for OF initiation, and throughout lagging-strand synthesis for a stabilizing effect of the (SSB)_35_ binding mode on the Pol III holoenzyme. *In vitro* experiments would be required to test these various hypotheses.

It is remarkable that simply doubling the amount of SSB has such a large effect on viability, even though the *ssb* gene is not known to be regulated and strong SSB over-production is deleterious [27]. The striking effects of increased SSB expression on viability suggest that the *in vivo* SSB-DNA complex equilibrium is finely balanced between binding modes and can be switched by different factors, including SSB concentration and SSB interactors. It is noteworthy that ψ is present only in a few bacterial species [14], and although χ is more widely distributed, it is not universal [1], [14], [15]. Since stabilization of Pol III can apparently be achieved by SSB interaction with a Pol III holoenzyme component other than HolC provided that the amount of SSB is doubled, bacteria which lack χψ may tune the stability of their Pol III holoenzyme via one of the minimal clamp-loader subunits, for example through SSB interactions that do not exist in *E. coli*, by a stronger interaction with DnaE, or by naturally expressing higher amounts of SSB than in *E. coli*, together with lower levels of competing SOS-induced polymerases.

## Materials and Methods

### Strains and constructions

Strains, plasmids and oligonucleotides used in this work are described in Table S1. New mutations were constructed by recombineering as described in [44] and using DY330 [45]. All other strains were constructed by P1 transduction. pAM-*holD* plasmid was cured prior to each experiment by growing cells in the absence of IPTG and plasmid-less colonies were isolated on minimal medium glucose casaminoacids (MM) plates. We checked that less than 5% of cells in the culture contained pAM-*holD* and less than 1% had acquired a suppressor mutation. All mutations introduced by P1 transduction were checked by PCR and all new mutations constructed by recombineering were checked by PCR and sequencing. *lexAind* and *recF* mutations were tested by measuring UV sensitivity. For *argE*::*ssb* construction, DY330 was transformed by electroporation with a *ssb*-Kan^R^ PCR fragment flanked by 50 bp of homology with *argE*. For *argE*::*ssb*ΔC5, DY330- *argE*::*ssb* (JJC5953) was transformed by electroporation with a PCR Cm^R^ fragment flanked by 50 bp homology with SSB C-ter sequence lacking the five last residues and 50 bp of homology with *argE*. In this construction the 5 last SSB residues are replaced by two stop codons and the Kan^R^ gene in *argE*::*ssb* is replaced by Cm^R^. For pAM-*holC* construction, *holC* was PCR amplified from the chromosome and cloned into the pAM34 vector after digestion with *Pst*I and *Hind*III; the resulting plasmid was verified by sequencing and complementation of the *holC* mutant. For pAM-*holCD* construction, *holC* was cloned from pAHM101 [12] in pAM-*holD* using *Ssp*1/*Bsa*B1 and *Xba*1; the resulting plasmid was verified by PCR and complementation of the *holC* mutant.

### Viability measurement

For spot assays, colonies formed in three days on MM at 30°C were suspended in MM salt medium. Serial 10-fold dilutions were then performed and 7 µl of dilutions 10^−2^ to 10^−6^ were spotted on three MM and three LB plates that were placed at 30°C, 37°C and 42°C. For pGB-*dinB* transformants, 10^−2^ to 10^−5^ dilutions of colonies obtained overnight on LB were used. In all cases, plates were scanned after 16–24 hours of incubation at 37°C and 42°C, and after 2 days of incubation at 30°C. Spot assays were performed at least twice for each strain. In addition, for viable strains colony forming units (cfu) were determined by plating appropriate dilutions of overnight MM cultures on MM and LB plates. The number of colony was counted after 16–24 hours of incubation for plates at 37°C and 42°C and after 48 hours of incubation for plates at 30°C. Each strain was tested at least three times and results confirmed the full viability observed in spot assays. Non-viable mutants could not be grown overnight and therefore the lack of viability was also checked by streaking several isolated plasmid-less colonies on LB and MM at 30°C, 37°C and 42°C.

#### Genome sequencing

Chromosomal DNA was extracted by SDS-proteinase K cell lysis, followed with phenol-chloroform, chloroform-isoamyl alcohol chromosome purification and isopropanol precipitation. 5 µg of DNA were used to generate a genomic library according to Illumina's protocol. The sequencing was performed at the High-throughput Sequencing Department of IMAGIF platform (https://www.imagif.cnrs.fr/plateforme-36-High-throughput_Sequencing_Platform.html, CNRS, Gif-Sur-Yvette, France). The library was sequenced paired-ends, with a read length of 36b, on a GAII_x_ to an expected depth of 50×. Sequence of the isogenic wild-type strain JJC40 was determined in parallel and reads from mutant and wild-type genomes were aligned using Illumina's package CASAVA-1.7.0. The point mutation was detected by Illumina's package CASAVA-1.7.0, and the 10 kb duplication using Illumina's software GenomeStudio.

#### β-galactosidase assays

β-galactosidase assays for measures of SOS induction were performed as described [46]. Since isolated JJC2067 colonies could not be propagated owing to the growth advantage of suppressor mutations, pAM-*holD* containing clones were grown overnight in MM lacking IPTG and diluted 50 fold in MM for the experiment. Cultures were checked for the loss of pAM-*holD* and for containing at most 1% suppressor mutations. The same procedure was used for JJC2068 as a control.

### Immunodetection assays

SSB and FtsZ proteins were detected in cell extracts using polyclonal chicken antibodies against SSB (gift from MM Cox, University of Wisconsin-Madison) and polyclonal rabbit antibodies against FtsZ (gift from J Camberg, National Institutes of Health, Bethesda, Maryland). Cell extracts were prepared from a fixed amount of exponentially growing cells. The cells were resuspended in 100 µl of Laemmli Buffer (Bio-Rad #161-0737) and incubated for 10 min at 100°C. Total cellular proteins were fractionated by SDS-PAGE on 12.5% gels and transferred to a Hybond Nitrocellulose membrane (Amersham) by electroblotting using a semidry transfer system. Immunodetection was carried out as described in the ECL^+^ kit (Amersham). Western blots were revealed using LAS-3000 FujiFilm and quantified with ImageQuant.

## Supporting Information

Figure S1 Δ*holD* colonies generate suppressed clones. Δ*holD* [pAM-*holD*] cultures propagated for 8 hours on MM devoid of IPTG, were about 90% cured of pAM-*holD*. Nevertheless, when Δ*holD* colonies were streaked out onto the same medium variable numbers of colonies with different sizes were observed, representing putative Δ*holD* suppressor clones (4 different streaks on MM at 30°C are shown, with a wild-type and a JJC2394 control).
(TIF)

Figure S2 Increased SSB amount in JJC2394 and *argE::ssb* strains compared to wild-type. Western blot analysis of SSB and FtsZ proteins was performed on extract from LB cultures grown at 37°C. Wild-type JJC40, Δ*holD* sup JJC2394, *argE::ssb* JJC6047. Samples were collected at OD_600 nm_ = 1. Band intensity was estimated using ImageQuant. For each strain, the intensity of the SSB band was divided by FtsZ and normalized to the wild-type strain; the intensity ratio between mutant and wild-type strains is indicated on the figure.
(TIF)

Figure S3 The capacity of Δ*holD* and Δ*holD* suppressed clones to form colonies is similar on MM (Figures 1, 3, 4) and on LB plates. Serial dilutions of colony suspensions used in Figures 1, 3, 4 were plated in parallel on three LB plates that were incubated overnight at 37°C or 42°C or for two days at 30°C. From top to bottom: wild-type, JJC2069; Δ*holD*, JJC2067 cured of pAM-*holD*; Δ*holD sup*, JJC2394; Δ*holD sup* Leu^+^, JJC6178; Δ*holD sup* dupΔ, JJC6217 cured of pAM-*holD*; Δ*holD*, 6050 cured of pAM-*holD*; Δ*holD argE::ssb*, JJC6110; Δ*holD lexAind*, JJC1524 cured of pAM-*holD*; Δ*holD lexAind argE::ssb*, JJC6077 cured of pAM-*holD*; *argE::ssb*Δ*C5*, JJC6162; Δ*holD*, JJC2067 cured of pAM-*holD*; Δ*holD argE::ssb*ΔC5, JJC6078 cured of pAM-*holD*; Δ*holD argE::ssb*, JJC6076 cured of pAM-*holD*; MG1655, JJC3523; MG1655 Δ*holD*, JJC6363 cured of pAM-*holD*; MG1655 Δ*holD lexAind*, JJC1524 cured of pAM-*holD*; MG1655 Δ*holD argE::ssb*, JJC6394 cured of pAM-*holD*; MG1655 Δ*holD lexAind argE::ssb* JJC6419.
(TIF)

Figure S4 The unknown mutation in JJC2394 does not affect homologous recombination or SOS induction. 5 µl drops of exponentially growing cultures (OD 0.3 to 0.4) serial dilutions (10^−1^ to 10^−5^) were plated on LB. One plate was not treated and one plate was UV-irradiated at 40 Joules/m^2^. Both plates were incubated over-night at 37°C. Wild-type, JJC1945; Δ*holD* sup, JJC2394; Δ*holD argE::ssb*, JJC6128; Δ*holD argE::ssb recF*, JJC6180; Δ*holD argE::ssb lexAind*, JJC6077 cured of pAM-*holD*. As expected *recF* inactivation confers a partial UV sensitivity to the Δ*holD argE::ssb* strain, while *lexAind* mutation (as *recA*) confers a strong UV sensitivity. JJC2394 (Δ*holD sup*) was as resistant to UV irradiation as wild-type or Δ*holD argE::ssb* cells, which confirms the absence of a mutation in *recFOR, recA* or *lexA* genes, or in any gene preventing homologous recombination.
(TIF)

Figure S5 pGB-*dinB* is lethal in a Δ*holD argE::ssb* context. Three top panels: serial dilutions spots on MM spectinomycin (7 µl of dilutions 10^−2^ to 10^−5^). Bottom panels: same dilutions on LB spectinomycin. Plates were incubated overnight at 37°C or 42°C and for two days at 30°C. JJC6110 (Δ*holD argE::ssb*) pGB-*dinB* transformants obtained on LB at 37°C could not be propagated on MM nor on LB at any temperature, in contrast to transformants containing the pGB2 vector or the plasmid expressing a mutant DinB protein affected for DnaN interaction. Viability of Δ*holD argE::ssb* [pGB-*dinB*] cells was restored by inactivation of the SOS response (*lexAind* mutation). Growth of the *lexA*Def mutant, which constitutively expresses the SOS response, was unaffected by pGB-*dinB*. Similar phenotypes were observed on MM and on LB.
(TIF)

Figure S6 Suppression of Δ*holC* and Δ*holD* Δ*holC* growth defects by *ssb* gene duplication. Serial dilutions of the colony suspensions used in Figures 6 were plated in parallel on three LB plates and incubated overnight at 37°C or 42°C or for two days at 30°C. From top to bottom: wild-type, JJC1945; Δ*holC*, JJC6469 cured of pAM-*holC*; Δ*holC argE::ssb*, JJC6476 cured of pAM-*holC*; Δ*holD* Δ*holC argE::ssb*, JJC6470 cured of pAM-*holC*; Δ*holC*, JJC6465 cured of pAM-*holCD*; Δ*holD* Δ*holC argE::ssb*, JJC6466 cured of pAM-*holCD*.
(TIF)

Table S1 Strains, plasmids and oligonucleotides.
(DOCX)

Table S2 pGB-*dinB* is lethal to Δ*holD sup* and Δ*holD argE::ssb* only when the SOS response is induced. JJC40 and JJC1945: wild-type; JJC2394: Δ*holD sup*; JJC6110: Δ*holD argE::ssb*; JJC6133: Δ*holD argE::ssb lexAind*; JJC6488: *lexA71*Def::Tn*5*. For each strain the number of transformants per ng of plasmid was calculated and normalized to the number of transformants per ng of pGB2 plasmid obtained on LB at 37°C (shown between parentheses). Transformants were counted after incubation at 37°C and 42°C overnight or after two days at 30°C. In one experiment pGB-*dinB* transformants appeared on MM at 37°C in JJC2394 or JJC6110, but they were not reproducibly obtained and similarly to the clones obtained on LB, they could not be propagated (Figure S4). np = did not propagate under any condition (cf Figure S4 spots of serially diluted JJC6110 [pGB-*dinB*] colonies). TS: impaired when propagated at 30°C and 37°C on LB and MM and strongly impaired when propagated at 42°C.
(DOCX)
