# Supplementary figures and images for: ssb Gene Duplication Restores the Viability of ΔholC and ΔholD Escherichia coli Mutants
Source: PLoS Genet. 2014 Oct 16;10(10):e1004719. doi: 10.1371/journal.pgen.1004719 (PMC4199511; doi:10.1371/journal.pgen.1004719)

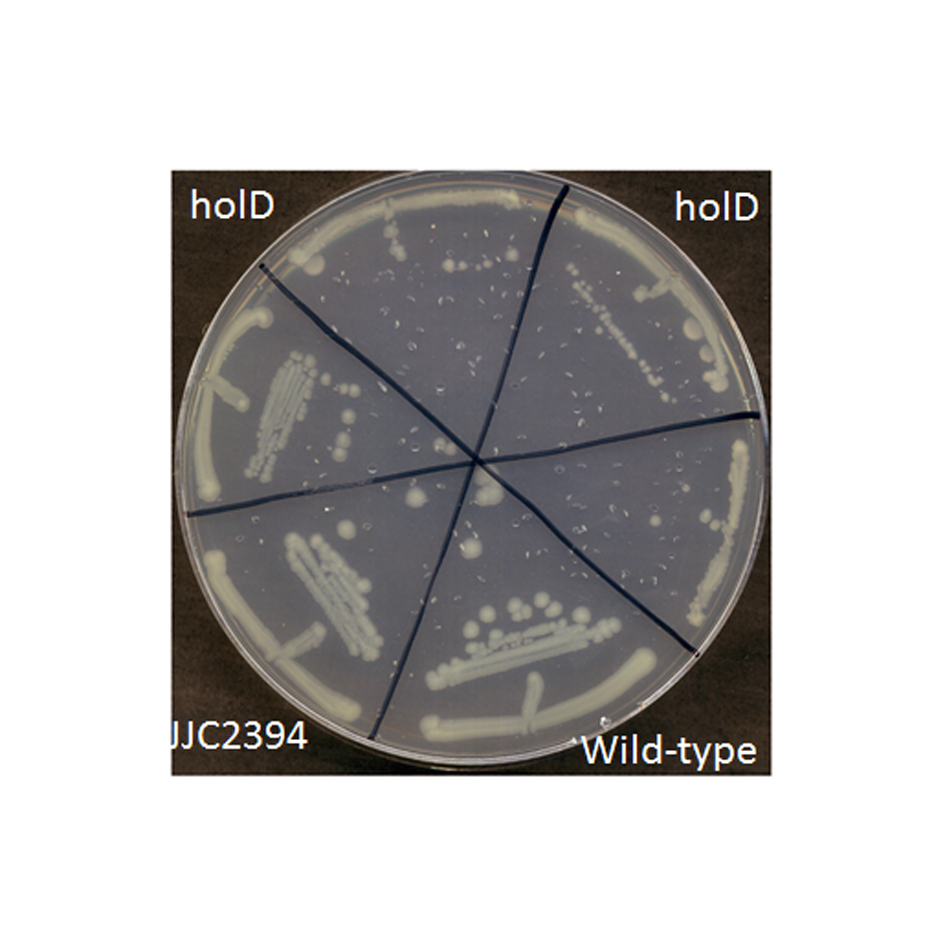

Supplement: Figure S1 — ΔholD colonies generate suppressed clones. ΔholD [pAM-holD] cultures propagated for 8 hours on MM devoid of IPTG, were about 90% cured of pAM-holD. Nevertheless, when ΔholD colonies were streaked out onto the same medium variable numbers of colonies with different sizes were observed, representing putative ΔholD suppressor clones (4 different streaks on MM at 30°C are shown, with a wild-type and a JJC2394 control). (TIF) [file pgen.1004719.s001.tif]

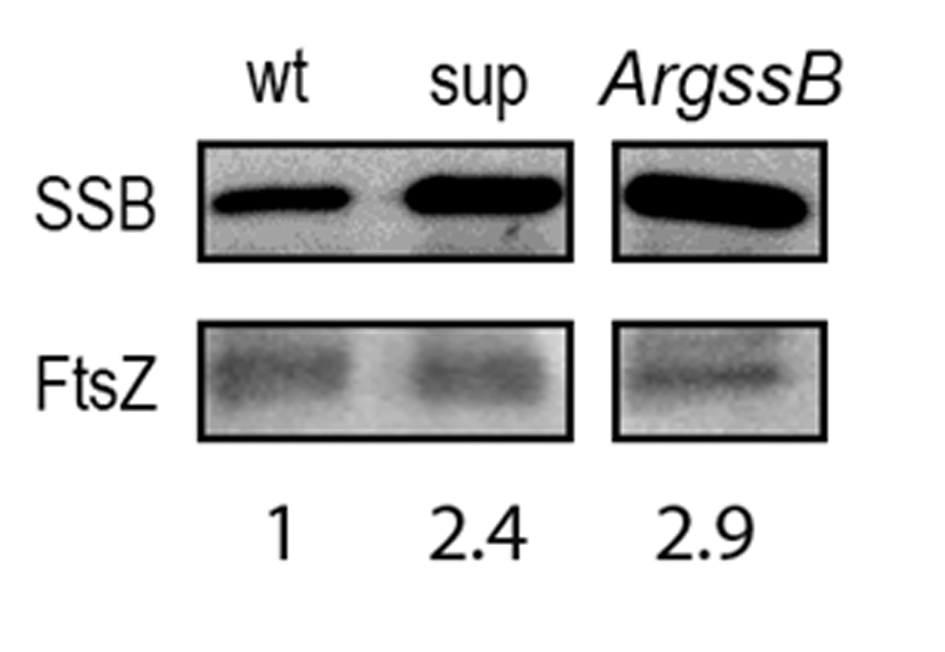

Supplement: Figure S2 — Increased SSB amount in JJC2394 and argE::ssb strains compared to wild-type. Western blot analysis of SSB and FtsZ proteins was performed on extract from LB cultures grown at 37°C. Wild-type JJC40, ΔholD sup JJC2394, argE::ssb JJC6047. Samples were collected at OD600 nm = 1. Band intensity was estimated using ImageQuant. For each strain, the intensity of the SSB band was divided by FtsZ and normalized to the wild-type strain; the intensity ratio between mutant and wild-type strains is indicated on the figure. (TIF) [file pgen.1004719.s002.tif]

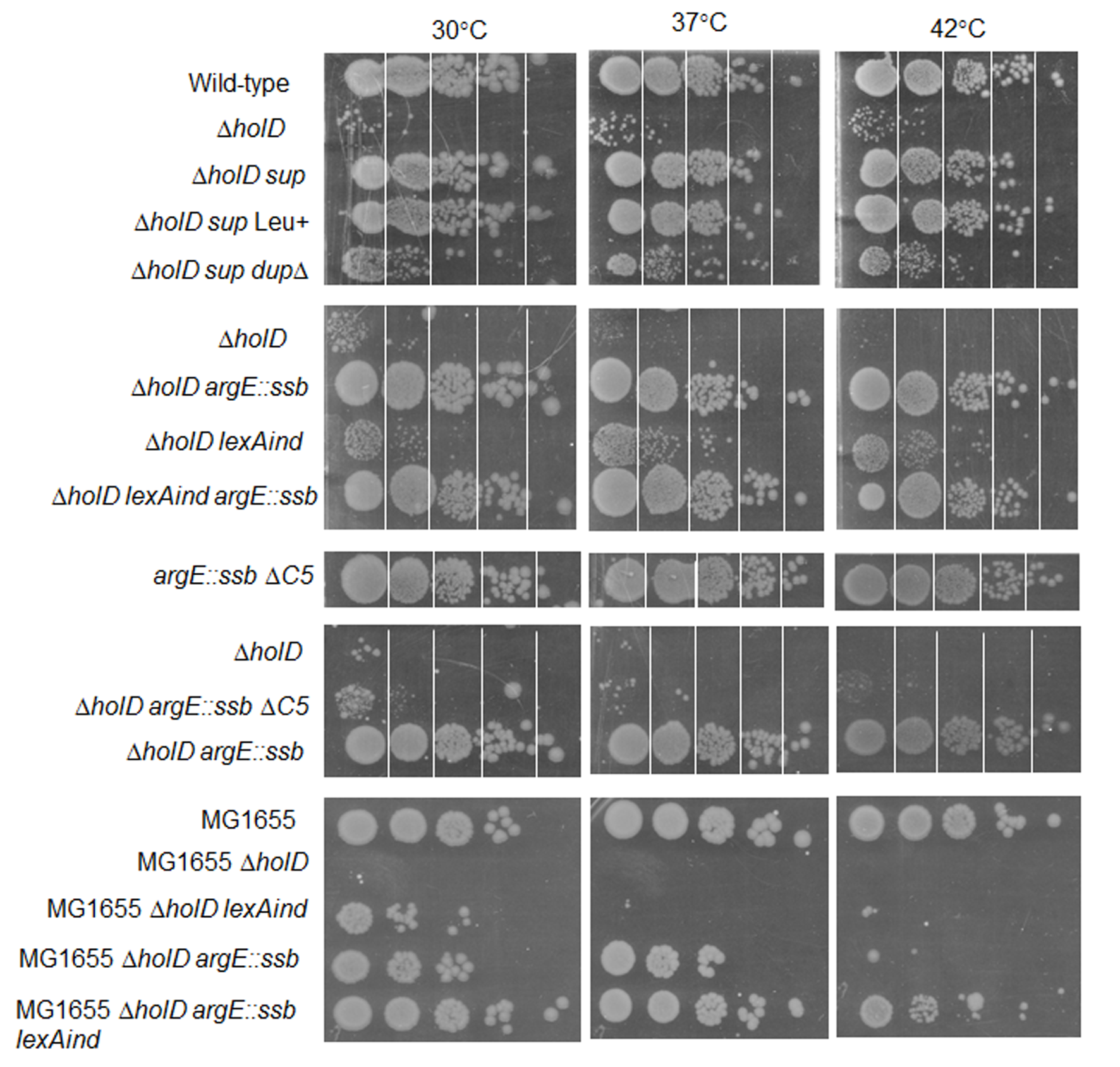

Supplement: Figure S3 — The capacity of ΔholD and ΔholD suppressed clones to form colonies is similar on MM (Figures 1, 3, 4) and on LB plates. Serial dilutions of colony suspensions used in Figures 1, 3, 4 were plated in parallel on three LB plates that were incubated overnight at 37°C or 42°C or for two days at 30°C. From top to bottom: wild-type, JJC2069; ΔholD, JJC2067 cured of pAM-holD; ΔholD sup, JJC2394; ΔholD sup Leu+, JJC6178; ΔholD sup dupΔ, JJC6217 cured of pAM-holD; ΔholD, 6050 cured of pAM-holD; ΔholD argE::ssb, JJC6110; ΔholD lexAind, JJC1524 cured of pAM-holD; ΔholD lexAind argE::ssb, JJC6077 cured of pAM-holD; argE::ssbΔC5, JJC6162; ΔholD, JJC2067 cured of pAM-holD; ΔholD argE::ssbΔC5, JJC6078 cured of pAM-holD; ΔholD argE::ssb, JJC6076 cured of pAM-holD; MG1655, JJC3523; MG1655 ΔholD, JJC6363 cured of pAM-holD; MG1655 ΔholD lexAind, JJC1524 cured of pAM-holD; MG1655 ΔholD argE::ssb, JJC6394 cured of pAM-holD; MG1655 ΔholD lexAind argE::ssb JJC6419. (TIF) [file pgen.1004719.s003.tif]

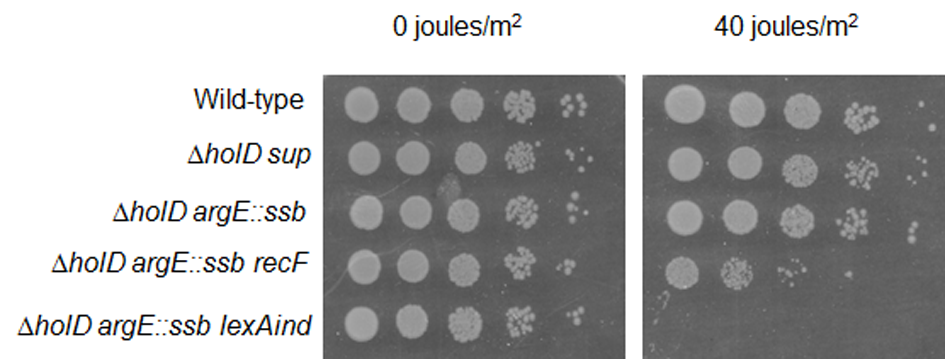

Supplement: Figure S4 — The unknown mutation in JJC2394 does not affect homologous recombination or SOS induction. 5 µl drops of exponentially growing cultures (OD 0.3 to 0.4) serial dilutions (10−1 to 10−5) were plated on LB. One plate was not treated and one plate was UV-irradiated at 40 Joules/m2. Both plates were incubated over-night at 37°C. Wild-type, JJC1945; ΔholD sup, JJC2394; ΔholD argE::ssb, JJC6128; ΔholD argE::ssb recF, JJC6180; ΔholD argE::ssb lexAind, JJC6077 cured of pAM-holD. As expected recF inactivation confers a partial UV sensitivity to the ΔholD argE::ssb strain, while lexAind mutation (as recA) confers a strong UV sensitivity. JJC2394 (ΔholD sup) was as resistant to UV irradiation as wild-type or ΔholD argE::ssb cells, which confirms the absence of a mutation in recFOR, recA or lexA genes, or in any gene preventing homologous recombination. (TIF) [file pgen.1004719.s004.tif]

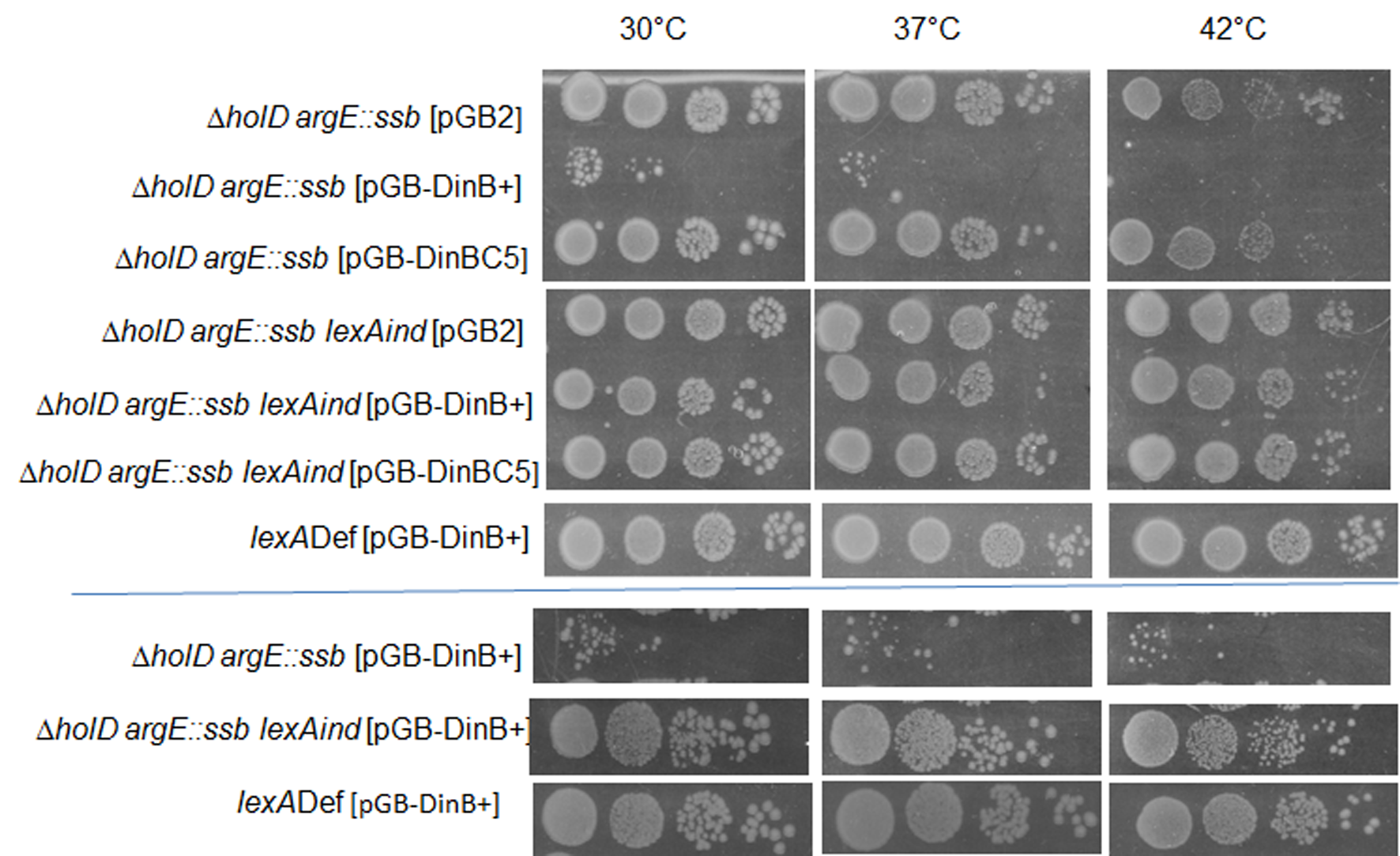

Supplement: Figure S5 — pGB-dinB is lethal in a ΔholD argE::ssb context. Three top panels: serial dilutions spots on MM spectinomycin (7 µl of dilutions 10−2 to 10−5). Bottom panels: same dilutions on LB spectinomycin. Plates were incubated overnight at 37°C or 42°C and for two days at 30°C. JJC6110 (ΔholD argE::ssb) pGB-dinB transformants obtained on LB at 37°C could not be propagated on MM nor on LB at any temperature, in contrast to transformants containing the pGB2 vector or the plasmid expressing a mutant DinB protein affected for DnaN interaction. Viability of ΔholD argE::ssb [pGB-dinB] cells was restored by inactivation of the SOS response (lexAind mutation). Growth of the lexADef mutant, which constitutively expresses the SOS response, was unaffected by pGB-dinB. Similar phenotypes were observed on MM and on LB. (TIF) [file pgen.1004719.s005.tif]

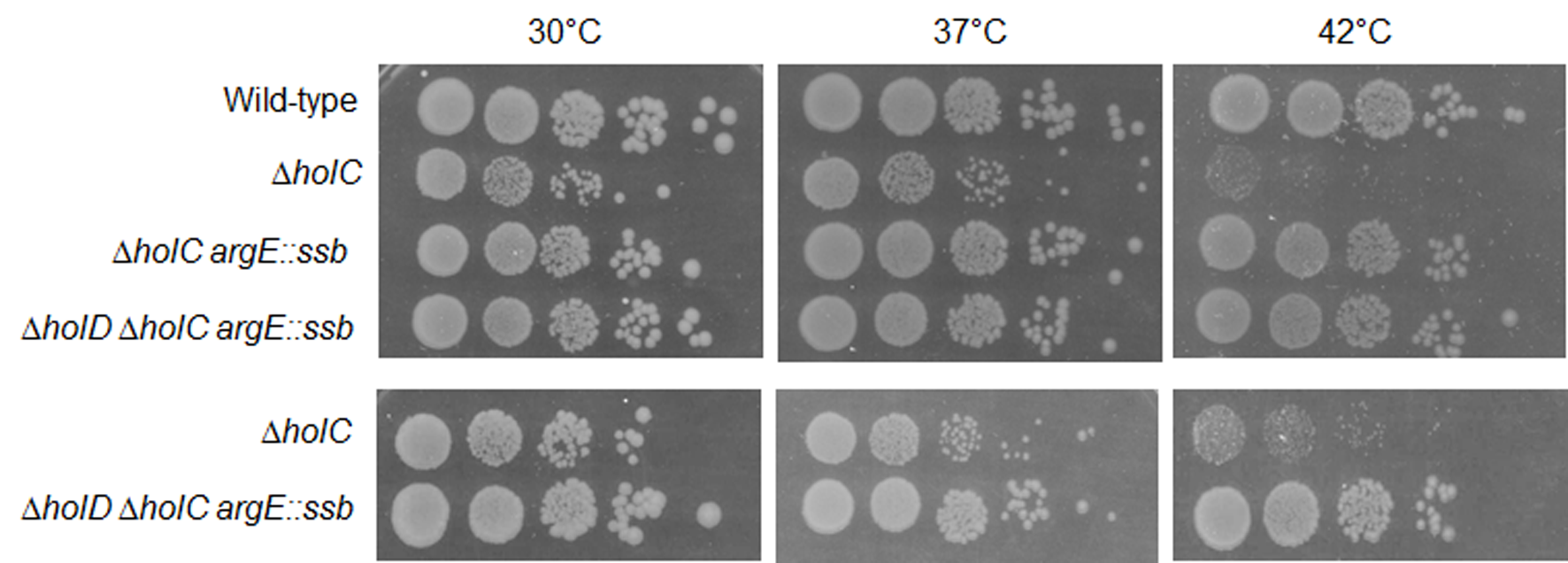

Supplement: Figure S6 — Suppression of ΔholC and ΔholD ΔholC growth defects by ssb gene duplication. Serial dilutions of the colony suspensions used in Figures 6 were plated in parallel on three LB plates and incubated overnight at 37°C or 42°C or for two days at 30°C. From top to bottom: wild-type, JJC1945; ΔholC, JJC6469 cured of pAM-holC; ΔholC argE::ssb, JJC6476 cured of pAM-holC; ΔholD ΔholC argE::ssb, JJC6470 cured of pAM-holC; ΔholC, JJC6465 cured of pAM-holCD; ΔholD ΔholC argE::ssb, JJC6466 cured of pAM-holCD. (TIF) [file pgen.1004719.s006.tif]
