# Supplementary material for: ssb Gene Duplication Restores the Viability of ΔholC and ΔholD Escherichia coli Mutants
Source: PLoS Genet. 2014 Oct 16;10(10):e1004719. doi: 10.1371/journal.pgen.1004719 (PMC4199511; doi:10.1371/journal.pgen.1004719)
Supplement: Table S1 — Strains, plasmids and oligonucleotides. (DOCX) [file pgen.1004719.s007.docx]

Table S1 Strains, plasmids and oligonucleotides.

| Strain | Relevant Genotype | | | Construction or origin | |
| --- | --- | --- | --- | --- | --- |
| JJC40 | AB1157 Pro^+^ Thr^+^ HsdR | | | [1] | |
| JJC523 | *recA441 sfiA11* Δ*lacI169 thi leu his arg ilv galK rpsl lexA*Def*71*::Tn*5* | | | Laboratory collection | |
| JJC1098 | *holC102*::Cm^R^ *zjg2086*::Kan^R^ *recA430* *srl*::Tn10 | | | RM4848 from Russell Maurer. Growth defects are suppressed by *recA430* that inactivates SOS (Russell Maurer, personal communication) | |
| JJC1489 | Δ*holD* ::Kan^R^ [pAM-*holD*] | | | [1] | |
| JJC1490 | Δ*holD* [pAM-*holD*] | | | [1] | |
| JJC1524 | Δ*holD* ::KanR *lexAind3 malE* ::Tn*10* [pAM-*holD*] | | | [1] | |
| JJC1945 | *sfiA11* | | | [1] | |
| JJC2067 | Δ*holD*  *sfiA*::MudAp*lacZ* lacZ::IR *zah281*::Tn*10* [pAM-*holD*] | | | [1] | |
| JJC2068 | Δ*holD*  *sfiA*::MudAp*lacZ* lacZ::IR *zah281*::Tn*10* [pAM-*holD*] *recF400*::Tn*5* | | | [1] | |
| JJC2069 | *sfiA*::MudAp*lacZ* lacZ::IR *zah281*::Tn*10* | | | [1] | |
| JJC2394 | Δ*holD*  *sfiA*::MudAp*lacZ* lacZ::IR *zah281*::Tn*10* sup | | | Spontaneous suppressor of JJC2067 cured of pAM-*holD* | |
| JJC3523 | MG1655 Δ*lacZ* | | | Laboratory collection | |
| JJC5684 | Δ*holD*  *sfiA*::MudAp*lacZ* lacZ::IR *zah281*::Tn*10* sup [pAM-*holD*] | | | JJC2394 transformed with pAM-*holD* (Cm^R^ expressed poorly) | |
| JJC5953 | DY330 *argE ::ssb-*Kan^R^ | | | *ssb-*KanR inserted by recombineering in *argE* | |
| JJC5954 | MG1655Δ*lacZ* Δ*attB* ::Spc^R^ *argE ::ssb-*Kan^R^ | | | JJC3524 [2] * P1 JJC5953 | |
| JJC6047 | *sfiA*::MudAp*lacZ* lacZ::IR *zah281*::Tn*10 argE ::ssb-*Kan^R^ | | | JJC2069 * P1 JJC5954 | |
| JJC6048 | Δ*holD* [pAM-*holD*] *sfiA*::Kan^R^ *pyrD* | | | JJC1490 * P1 *sfiA*::Kan^R^ *pyrD* | |
| JJC6050 | Δ*holD*  *sfiA11* [pAM-*holD*] | | | JJC6048 * P1 *sfiA11* Pyr^+^ | |
| JJC6056 | Δ*holD* *sfiA11* [pAM-*holD*] *argE ::ssb-*Kan^R^ | | | JJC6050 * P1 JJC5954 | |
| JJC6060 | Δ*holD*  *sfiA*::MudAp*lacZ* lacZ::IR *zah281*::Tn*10* sup *recF400*::Tn*5* | | | JJC2394 * P1 *recF400* ::Tn*5* | |
| JJC6062 | DY330 *argE ::ssb*ΔC5*-*Cm^R^ | | | *ssb*ΔC5*-*Cm^R^ inserted into JJC5953 *argE* by recombineering | |
| JJC6073 | *argE ::ssb*ΔC5*-*Cm^R^ *sfiA11* | | | JJC1945 * P1 JJC6062 | |
| JJC6076 | Δ*holD* *sfiA11* [pAM-*holD*] *argE ::ssb* | | | JJC6056 with Kan^R^ adjacent to *argE ::ssb* excised by FLP recombination | |
| JJC6077 | Δ*holD* *sfiA11* [pAM-*holD*] *argE ::ssb-*Kan^R^ *lexAind3 mal*::Cm^R^ | | | JJC6056 * P1 *lexAind3 mal*::Tn*9* | |
| JJC6078 | Δ*holD*  *sfiA11* [pAM-*holD*] *argE ::ssb*ΔC5*-*Cm^R^ | | | JJC6050 * P1 JJC6062 | |
| JJC6095 | Δ*holD* *sfiA11* [pAM-*holD*] *argE ::ssb lexAind3 malF3180::*Kan^R^ | | | JJC6076 * P1 *lexAind3 malF3180::*Kan^R^ | |
| JJC6110 | Δ*holD* *sfiA11* *argE ::ssb* | | | JJC6076 cured of pAM-*holD* | |
| JJC6117 | Δ*holD* *sfiA11* [pAM-*holD*] *argE ::ssb lacZ6373::*IS*1223* (Cm^R^) | | | JJC6076 * P1 *lacZ6373::*IS*1223* (Cm^R^) | |
| JJC6122 | Δ*holD* *sfiA11* *argE ::ssb lacZ6373::*IS*1223* (Cm^R^) | | | JJC6117 cured of pAM-*holD* | |
| JJC6128 | Δ*holD* *sfiA*::MudAp*lacZ* *argE ::ssb lacZ6373::*IS*1223* (Cm^R^) | | | JJC6122 * P1 *sfiA*::MudAp*lacZ* | |
| JJC6133 | Δ*holD* *sfiA11* *argE ::ssb lexAind3 malF3180::*Kan^R^ | | | JJC6095 cured of pAM-*holD* | |
| JJC6147 | *argE ::ssb*ΔC5*-*Cm^R^ *sfiA11 pflD::*Kan^R^ | | | JJC6073 *P1 CAG18560 [3] | |
| JJC6161 | Δ*holD*  *sfiA*::MudAp*lacZ* lacZ::IR *zah281*::Tn*10* [pAM-*holD*] *argE ::ssb-*Kan^R^ | | | JJC2067 * P1 JJC5954 | |
| JJC6162 | *sfiA*::MudAp*lacZ* lacZ::IR *zah281*::Tn*10* [pAM-*holD*] *argE ::ssb*ΔC5*-*CmR *pflD* ::Kan^R^ | | | JJC2069 * P1 JJC6147 | |
| JJC6174 | Δ*holD*  *sfiA*::MudAp*lacZ* lacZ::IR *zah281*::Tn*10* sup [pAM-*holD*] | | | JJC2394 * pAM-*holD* | |
| JJC6178 | Δ*holD*  *sfiA*::MudAp*lacZ* lacZ::IR *zah281*::Tn*10* sup [pAM-*holD*] | | | JJC6174 * P1 JJC5954 | |
| JJC6180 | Δ*holD* *sfiA*::MudAp*lacZ* *argE ::ssb lacZ::*CmR *recF400*::Tn*5* *zid*::Tn*10* | | | JJC6128 * P1 *recF400* ::Tn*5* *zid*::Tn*10* | |
| JJC6190 | Δ*holD*  *sfiA*::Kan^R^ *pyrD* lacZ::IR *zah281*::Tn*10* sup | | | JJC2394 * P1 *sfiA* ::KanR *pyrD* | |
| JJC6198 | Δ*holD*  *sfiA11* lacZ::IR *zah281*::Tn*10* sup | | | JJC6190 * P1 *sfiA11* PyrD^+^ | |
| JJC6203 | Δ*holD*  *sfiA11* lacZ::IR *zah281*::Tn*10* sup *argE ::ssb-*Kan^R^ | | | JJC6198 * P1 JJC5954 | |
| JJC6212 | Δ*holD*  *sfiA11* lacZ::IR *zah281*::Tn*10* sup *argE ::ssb* | | | JJC6203 with Kan^R^ adjacent to *argE ::ssb* excised by FLP recombination | |
| JJC6216 | Δ*holD*  *sfiA*::MudAp*lacZ* lacZ::IR *zah281*::Tn*10* sup Δdup *argE ::ssb* | | | JJC6212 * P1 *sfiA*::MudAp*lacZ* | |
| JJC6217 | Δ*holD*  *sfiA*::MudAp*lacZ* lacZ::IR *zah281*::Tn*10* sup [pAM-*holD*] sup Δdup | | | JJC2394 * P1 CAG12019 [3] | |
| JJC6351 | MG1655 Δ*lacZ* [pAM-*holD*] | | | JJC3523 [2] transformed with pAM-*holD* | |
| JJC6356 | MG1655 Δ*lacZ* [pAM-*holD*] Δ*holD* ::Kan^R^ | | | JJC6351 * P1 JJC1489 | |
| JJC6363 | MG1655 Δ*lacZ* [pAM-*holD*] Δ*holD* ::Kan^R^ *sfiA*::MudAp*lacZ* | | | JJC6356 * P1 *sfiA*::MudAp*lacZ* | |
| JJC6382 | MG1655 Δ*lacZ* [pAM-*holD*] Δ*holD* | | | JJC6356 with Kan^R^ excised from *holD* by FLP recombination | |
| JJC6392 | MG1655 Δ*lacZ* [pAM-*holD*] Δ*holD* *argE ::ssb-*Kan^R^ | | | JJC6382 * P1 JJC5954 | |
| JJC6394 | MG1655 Δ*lacZ* [pAM-*holD*] Δ*holD* *argE ::ssb-*Kan^R^ *sfiA*::MudAp*lacZ* | | | JJC6392 * P1 *sfiA*::MudAp*lacZ* | |
| JJC6412 | *sfiA11* *argE ::ssb-*Kan^R^ | | | JJC1945 * P1 JJC5954 | |
| JJC6420 | MG1655 Δ*lacZ* [pAM-*holD*] Δ*holD* *argE ::ssb-*KanR *lexAind3 malF3089*::Tn*10* | | | JJC6394 * P1 *lexAind3 malF3089*::Tn*10* | |
| JJC6443 | *sfiA11* [pAM-*holCD*] | | | JJC1945 transformed with pAM-*holCD* | |
| JJC6454 | *sfiA11* Δ*holD* *argE ::ssb* [pAM-*holCD*] | | | JJC6110 transformed with pAM-*holCD* | |
| JJC6462 | *sfiA11* [pAM-*holC*] | | | JJC1945 transformed with pAM-*holC* | |
| JJC6463 | *sfiA11* Δ*holD* *argE ::ssb* [pAM-*holC*] | | | JJC6110 transformed with pAM-*holC* | |
| JJC6464 | *sfiA11* *argE ::ssb* | | | JJC6412 with Kan^R^ excised by FLP recombination | |
| JJC6465 | *sfiA11* *holC102*::Cm^R^ *zjg2086*::Kan^R^ [pAM-*holCD*] | | | JJC6443 * P1 JJC1098 | |
| JJC6466 | *sfiA11* Δ*holD* *argE ::ssb holC102*::Cm^R^ *zjg2086*::Kan^R^ [pAM-*holCD*] | | | JJC6454 * P1 JJC1098 | |
| JJC6467 | *sfiA11* *argE ::ssb* [pAM-*holC*] | | | JJC6464 transformed with pAM-*holC* | |
| JJC6468 | *sfiA11* *argE ::ssb* [pAM-*holCD*] | | | JJC6464 transformed with pAM-*holCD* | |
| JJC6469 | *sfiA11 holC102*::CmR *zjg2086*::Kan^R^ [pAM-*holC*] | | | JJC6462 * P1 JJC1098 | |
| JJC6470 | *sfiA11* Δ*holD* *argE ::ssb holC102*::Cm^R^ *zjg2086*::Kan^R^ [pAM-*holC*] | | | JJC6463 * P1 JJC1098 | |
| JJC6476 | *sfiA11* *argE ::ssb holC102*::Cm^R^ *zjg2086*::Kan^R^ [pAM-*holC*] | | | JJC6467 * P1 JJC1098 | |
| JJC6477 | *sfiA11* *argE ::ssb holC102*::Cm^R^ *zjg2086*::Kan^R^ [pAM-*holCD*] | | | JJC6468 * P1 JJC1098 | |
| JJC6488 | *sfiA*::MudAp*lacZ* lacZ::IR *zah281*::Tn*10* sup *lexA*Def71::Tn*5* | | | JJC2069 * P1 JJC523 | |
| Plasmids | | | | | |
| pAM34 | | pBR322-derived plasmid where the RNA primer original promoter was replaced by the *lac* promoter; carries Ap^R^, Spec^R^ and *lacI* genes | | | [4] |
| pAM-*holD* | | pAM34 carrying the *holD* gene cloned in *Eco*RI; Ap^R^, Spec^R^ | | | [1] |
| pAM-*holC* | | pAM34 carrying the *holC* gene cloned in *Bam*HI-*Hind*III; Ap^R^ | | | This work |
| pAM-*holCD* | | pAM-*holD* carrying the *holC* gene cloned in *Bsa*BI-*Xba*I; Ap^R^, Spec^R^ | | | This work |
| pGB-*dinB* | | pGB2-derived plasmid carrying the wild-type *dinB* gene | | | [5] |
| pGB-*dinB*ΔC5 | | pGB2-derived plasmid carrying the *dinB* gene deleted for the 5 terminal amino acids | | | [5] |
| Oligonucleotides | | | | | |
| Number | | | Sequence | | Use |
| #73 | | | GGAATTCCCGAACTTCGATCTTATCC | | check 10kb duplication |
| #84 | | | GCCGAGGAGGTTAAAGGTGA | | check 10kb duplication |
| #87 | | | TACTCGCGATGGAGATGATG | | check 10kb duplication |
| #88 | | | CATTAAAGCGCTGCTAACCC | | check 10kb duplication |
| #92 | | | TTGATAAACCGTGTTTCCAGATATTCATCAGGTTGATGAGCCTGATTAATCCATATGAATATCCTCCTTA | | construction *argE*::*ssb*ΔC5 |
| #93 | | | AATTACCGCCATTTATCGAGATTTACCGCGCTCTGATTGCCACCTTTCCCGGATTAAACG | | construction *argE*::*ssb* |
| # 94 2 | | | CTAGAAAGTATAGGAACTTCGAAGCAGCTCCAGCCTACACATCAGAACGGAATGTCATCATC | | construction *argE*::*ssb* |
| #95 | | | GCGCCGTCTAACGAGCCGCCGATGGACTTTGATGATGACATTCCGTTCTGATGTGTAGGCTGGAGCTGCTTCG | | construction *argE*::*ssb* |
| #109 | | | GCTCTGATTGCCACACCTTC | | check *argE*::*ssb* and *argE*::*ssb*ΔC5 |
| #110 | | | CGCGGGTGGGCTTGATAAAC | | check *argE*::*ssb* and *argE*::*ssb*ΔC5 |
| #146 | | | TCTTCGGCCAGTAGTAAATCAGC | | check Δ*holD*::kan |
| #147 | | | GTAATTGCGGCGAATCGTCG | | check Δ*holD*::kan |
| #145 | | | GCAGTCCGCTCCGGCAGCGCCGTCTAACGAGCCGCCGATGGACTTTGATTGATTTAAATGGCGCGCCTTACGC | | construction *argE*::*ssb*ΔC5 |
| #153 | | | TCGAGATTTACCGCGCTCTG | | check *argE*::*ssb* and *argE*::*ssb*ΔC5 |
| #420 | | | CGCGCATGGCGCTTACCGCTGG | | check Δ*holC*::cm |
| #421 | | | CGAACAGCCGCTTTACGAGCAC | | check Δ*holC*::cm |

Unless otherwise indicated, all strains derive from JJC40
