# Supplementary material for: ssb Gene Duplication Restores the Viability of ΔholC and ΔholD Escherichia coli Mutants
Source: PLoS Genet. 2014 Oct 16;10(10):e1004719. doi: 10.1371/journal.pgen.1004719 (PMC4199511; doi:10.1371/journal.pgen.1004719)
Supplement: Table S2 — pGB-dinB is lethal to ΔholD sup and ΔholD argE::ssb only when the SOS response is induced. JJC40 and JJC1945: wild-type; JJC2394: ΔholD sup; JJC6110: ΔholD argE::ssb; JJC6133: ΔholD argE::ssb lexAind; JJC6488: lexA71Def::Tn5. For each strain the number of transformants per ng of plasmid was calculated and normalized to the number of transformants per ng of pGB2 plasmid obtained on LB at 37°C (shown between parentheses). Transformants were counted after incubation at 37°C and 42°C overnight or after two days at 30°C. In one experiment pGB-dinB transformants appeared on MM at 37°C in JJC2394 or JJC6110, but they were not reproducibly obtained and similarly to the clones obtained on LB, they could not be propagated (Figure S4). np = did not propagate under any condition (cf Figure S4 spots of serially diluted JJC6110 [pGB-dinB] colonies). TS: impaired when propagated at 30°C and 37°C on LB and MM and strongly impaired when propagated at 42°C. (DOCX) [file pgen.1004719.s008.docx]

Table S2: pGB-*dinB* is lethal to Δ*holD sup* and Δ*holD argE::ssb* only when the SOS response is induced.

| 1 ng plasmid | pGB2 | | | | | pGB-*dinB* | | | | | pGB-*dinB*ΔC5 | | | | |
| --- | --- | --- | --- | --- | --- | --- | --- | --- | --- | --- | --- | --- | --- | --- | --- |
| Strain  JJC | 40/ 1945 | 2394 | 6110 | 6133 | 6488 | 40/  1945 | 2394 | 6110 | 6133 | 6488 | 40/  1945 | 2394 | 6110 | 6133 | 6488 |
| MM 37°C | 1 | 1 | 1.4 | 0.7 | 0.8 | 0.6 | <0.04 | <0.04 | 0.8 | 0.9 | 0.8 | 1.3 | 1 | 1.2 | 0.8 |
| MM 42°C | 1.4 | 1.3 | 0.9 | 1.1 | 1.1 | 1.9 | <0.04 | <0.04 | 1 | 1.1 | 1 | 1.5 | 1.3 | 1 | 1.2 |
| LB 30°C | 0.7 | 0.7 | 1.3 | 0.7 | 0.7 | 1 | 0.8 ^TS^ | 0.7 ^TS^ | 0.9 | 0.7 | 0.6 | 0.7 | 0.9 | 0.7 | 0.7 |
| LB 37°C | 1 (165) | 1 (109) | 1 (128) | 1 (97) | 1  (91) | 0.7 | 1 ^TS^ | 1 ^np^ | 0.9 | 1 | 0.6 | 1 | 0.9 | 1 | 0.8 |
| LB 42°C | 0.8 | 1.2 | 1 | 0.9 | 1 | 1.2 | 0.9 ^np^ | 1 ^np^ | 0.9 | 1 | 0.7 | 1 | 1.1 | 1 | 0.8 |

JJC40 and JJC1945: wild-type; JJC2394: Δ*holD sup*; JJC6110: Δ*holD argE::ssb*; JJC6133: Δ*holD argE::ssb* *lexAind*; JJC6488: *lexA71*Def::Tn*5*

For each strain the number of transformants per ng of plasmid was calculated and normalized to the number of transformants per ng of pGB2 plasmid obtained on LB at 37°C (shown between parentheses). Transformants were counted after incubation at 37°C and 42°C overnight or after two days at 30°C. In one experiment pGB-*dinB* transformants appeared on MM at 37°C in JJC2394 or JJC6110, but they were not reproducibly obtained and similarly to the clones obtained on LB, they could not be propagated (Figure S4). np = did not propagate under any condition (cf Figure S4 spots of serially diluted JJC6110 [pGB-*dinB*] colonies). TS: impaired when propagated at 30°C and 37°C on LB and MM and strongly impaired when propagated at 42°C.
